# A Retrospective Analysis of ADAMTS13 Enzymatic Activity Results in a Population With Thrombotic Thrombocytopenic Purpura Disease in the Arabian Gulf and Colombia (APOLO Study)

**DOI:** 10.7759/cureus.103823

**Published:** 2026-02-18

**Authors:** Sherif Edris, Yahia Aktham, Ahmed Mekky, Mohamed Z Chouikrat, Hadia B Al Mahdi, Mahmoud Younis, María A Cortés, Maria F Vargas, Monica Fahmy

**Affiliations:** 1 Biological Sciences, Faculty of Science, King Abdulaziz University, Jeddah, SAU; 2 Bionanoscience Research, Centre of Excellence for Bionanoscience Research, King Abdulaziz University, Jeddah, SAU; 3 Genetics, Ain Shams University, Cairo, EGY; 4 Research and Development, Al Borg Medical Laboratories, Jeddah, SAU; 5 Medical Affairs, Sanofi Greater Gulf, Dubai, ARE; 6 Medical Affairs, Sanofi Greater Gulf, Bogotá, COL

**Keywords:** adamts-13 deficiency, arabian gulf, colombia, thrombotic thrombocytopenic purpura, ttp diagnosis

## Abstract

Little is known about the thrombospondin type-1 motif, member 13 (ADAMTS-13) enzymatic activity in subjects with suspected/confirmed thrombotic thrombocytopenic purpura (TTP) in the Arabian Gulf and Colombia. The retrospective analysis of ADAMTS13 enzymatic activity results in a population with thrombotic thrombocytopenic purpura disease in the Arabian Gulf and Colombia (APOLO study) aimed to report on ADAMTS-13 enzymatic activity, subject demographics, and the turnaround time for results. This study used laboratory analyses of ADAMTS-13 enzymatic activity in blood samples from subjects with suspected/confirmed TTP in the Arabian Gulf (N=235) and in Colombia (N=155). The primary objective was to report ADAMTS-13 enzymatic activity levels and distribution in categories: severe deficiency <10% (TTP diagnosis), borderline activity 10-20%, and no deficiency >20%. Descriptive statistics were performed using SAS 9.4 (SAS Institute Inc., North Carolina, USA). Among the 390 samples, the average ADAMTS-13 activity level was 42.8%±31.1. Severe deficiency (ADAMTS-13 activity <10%, 1.7%±1.9, indicative of TTP) was found in 91 samples (23.3%) and borderline ADAMTS-13 activity (15.4%±3.0) in 17 (4.4%) samples. The median age among subjects with TTP was 39 years. The average turnaround time for results was 4.4±5.9 days, with a median of 2.0 days. The average turnaround time was significantly longer for samples from the Arabian Gulf (6.5±6.9 days) compared to those from Colombia (1.2±0.8 days, Wilcoxon/Mann-Whitney W-value=18507.5, P<0.001). In conclusion, the median age of subjects with TTP in this study matched the age reported in the literature. The turnaround time is a global concern, as it can lead to delayed confirmatory results and potentially life-saving treatment. In Colombia, the turnaround time considerably benefits TTP management, underscoring the necessity to determine reasons behind occasional result turnaround delays in the Arabian Gulf.

## Introduction

Thrombotic thrombocytopenic purpura (TTP) is a type of thrombotic microangiopathy (TMA) that results from systemic microvascular thrombosis, leading to thrombocytopenia, hemolytic anemia, and organ failure [1]. TTP is caused by a severe deficiency of ADAMTS-13 (a disintegrin and metalloproteinase with a thrombospondin type 1 motif, member 13), causing the accumulation of ultra-large von Willebrand factor multimers, which bind to platelets and induce aggregation [2]. In normal circulation, ADAMTS-13 prevents the unwanted development of thrombi, and in hemostasis, it prevents the excessive growth of platelet plugs [3]. While TTP may be congenital (hereditary), 95% of cases remain acquired (immune-mediated) [1,4]. Both hereditary and immune TTP are associated with ADAMTS-13 activity deficiency [5]. Immune (acquired) TTP remains a rare disease (one to two cases per million, globally), but anti-ADAMTS-13 inhibitory autoantibodies against the spacer domain are present in 97%-100% of patients with acquired TTP [3]. Diagnosis usually occurs at the average age of 40, and TTP seems to affect female patients more frequently [6,7]. Clinical manifestations depend on the affected tissue and typically begin after age 30. Possible triggers include inflammation, infection, sepsis, surgery, medication, and pregnancy [4,8,9]. Risk factors include obesity, black ethnicity, female gender, and expression of the human leukocyte antigen class II histocompatibility, D-related beta chain (HLA-DRB1*11) antigen [4,9]. Recently, TTP has been reported with cases of non-ST-segment elevation myocardial infarction (non-STEMI), STEMI, and ischemic stroke [10-12]. The platelet count; combined hemoLysis variable; absence of active cancer; absence of stem-cell or solid-organ transplant; mean corpuscular volume; international normalised ratio; creatinine (PLASMIC) and French TTP scores can help guide clinical decisions when ADAMTS-13 testing is not immediately available [13,14], but the only unequivocal marker for confirming TTP is ADAMTS-13 activity deficiency and the presence of anti-ADAMTS-13 autoantibodies [3,5]. TTP is associated with ADAMTS-13 activity levels below 10% (although as many as 40% of patients with clinically suspected TTP have ADAMTS-13 activity levels greater than 10%) [15]. Normal plasma ADAMTS-13 antigen levels range from 740 to 1420 ng/ml (median 1080 ng/ml), resulting in an ADAMTS-13 activity-to-antigen ratio of 0.48 to 1.68 U/mg [16]. Several assays have been developed (antigen, antibody, and activity assays) with varying turnaround times [3,9,17,18].

However, diagnosis mostly relies on the clinical picture, and treatment is based on three pathophysiological axes: replenishment of ADAMTS-13 levels, immunomodulation, and inhibition of von Willebrand factor-platelet interaction [3,17]. While treatment with plasma exchange and immunosuppressant agents is life-saving, complications might arise [19,20], and relapse remains unpredictable and frequent [21]. In 2019, the US Food and Drug Administration approved caplacizumab, an anti-von Willebrand factor humanized single-variable-domain immunoglobulin, for the treatment of TTP in adult patients [22], following multiple clinical trials and case reports [23-29]. According to the International Society on Thrombosis and Haemostasis (ISTH), treatment is maintained as long as ADAMTS-13 activity levels are <10%, and stopped when they reach the threshold of 20% [30]. Immunosuppression with corticosteroids and rituximab has also proven beneficial for patients with TTP, as comprehensively reviewed [31]. With various therapeutic options currently available, timely diagnosis is vital because TTP is a medical emergency with fatal outcomes in 90% of cases left untreated [1,3,4,32,33].

Little is known about the assessment of ADAMTS-13 deficiency as a diagnostic tool for TTP in the Arabian Gulf region [34] and in Colombia, where a cost-consequence study underscored the importance of early diagnosis and treatment from a third-party payer standpoint [35]. In both areas, the kit is rarely available in-house, resulting in a turnaround time of approximately three weeks. This study analyzed laboratory results for ADAMTS-13 activity from blood samples collected as part of routine practice to gain insight into TTP diagnosis, including the distribution of suspected cases across ADAMTS-13 enzymatic activity levels and the turnaround time for results in the Arabian Gulf or Colombia.

## Materials and methods

Study design and samples

The retrospective analysis of ADAMTS13 enzymatic activity results in a population with thrombotic thrombocytopenic purpura disease in the Arabian Gulf and Colombia (APOLO study) was a multinational, retrospective study based on secondary analysis of laboratory data of plasma samples obtained in routine clinical practice from subjects with suspected/confirmed TTP. Blood samples, obtained outside of the scope of the current study, were analyzed for ADAMTS-13 enzymatic activity in a clinical laboratory. Sample results were from the following countries: Saudi Arabia, United Arab Emirates, Kuwait, and Colombia. Data from the three Arabian Gulf countries were pooled together and reported as one population for the Arabian Gulf. Blood samples were obtained and processed between 2021 and 2023. Laboratory results eligible for inclusion in this study had to come from adult male or female subjects over the age of 18 years, presenting with a clinical profile of TMA and suspected TTP, at the discretion of the treating physician, to be explicitly provided for TTP diagnostic purposes and to be either newly obtained or recently archived from subjects with suspected TTP and/or as a follow-up of confirmed TTP. No blood samples were drawn for the purposes of this study, which only analyzed laboratory data in aggregate, anonymous form.

ADAMTS-13 enzymatic activity assessment

Independent of the study, laboratories in Saudi Arabia (evaluating samples from the Arabian Gulf) and in Colombia used the following assay kits to evaluate ADAMTS-13 enzymatic activity. In the Arabian Gulf laboratory, a detailed protocol for handling plasma samples and assessing ADAMTS-13 enzymatic activity (5450701 TECHNOZYM® ADAMTS-13 Activity ELISA, Technoclone, Austria) was followed, according to the manufacturer’s instructions. In Colombia, the protocol for processing and handling samples to measure ADAMTS-13 enzyme activity levels was applied according to the manufacturer’s instructions. ADAMTS-13 activity was quantified using the HemosIL AcuStar ADAMTS13 Activity Assay (Werfen, USA). Notably, no laboratory tests were performed explicitly for this study, and all participating laboratories use validated assays to determine ADAMTS-13 enzymatic activity level.

Ethical considerations

The retrospective design and analysis of existing aggregated anonymized data did not require informed consent from subjects. Ethical approval for the study conducted in the Arabian Gulf was obtained from the Unit of Biomedical Ethics at Al Borg Diagnostics Laboratory (Institutional Review Board Approval Number No. 11/23) on July 24, 2023. Given the retrospective nature of the study and the use of an aggregate dataset, no ethical approval was required in Colombia.

Objectives

The primary objective was to determine the distribution of samples across enzymatic activity ranges: severe deficiency <10% (TTP diagnosis), borderline activity 10-20%, and no deficiency >20% (absence of TTP). Samples were collected and analyzed between 2021 and 2023, according to the ISTH [30]. Additionally, the data were also classified into two categories based on the 10% activity level cutoff: ADAMTS-13 enzymatic activity <10% and ≥10%. This classification was used to demarcate the proportion of subjects with severe deficiency (i.e., a TTP diagnosis) from those who do not have TTP.

Other objectives included (1) describing the demographic characteristics of the included population, (2) reporting the turnaround time from collection to results (both locally and internationally), and (3) mapping collection sites, regions, and countries.

Study size

The sample size was not based on formal statistical assumptions (convenience sampling as per available data). This exploratory study analyzed the results of all plasma samples collected over the two-year period from 2021 to 2023 in four different countries during regular referrals, as per the laboratory's routine practice, and not specifically for this study. It was expected to analyze sample results from up to 140 subjects (100 in the Arabian Gulf and 40 in Colombia). A total of 100 subjects was deemed sufficient for the analysis. If 100 eligible subjects are identified, it is possible to estimate an analysis rate of 50% within a 95% confidence interval (CI) and with 10% precision. This sample would allow a descriptive analysis of diagnostic and clinical characteristics among pre-defined subgroups as well. However, a much larger sample size was included for analysis (sample results from 390 subjects). Although samples were included based on convenience sampling, the laboratories providing the data are leading laboratories in the two regions and are expected to cover the vast majority of cases evaluated for TTP.

Statistical considerations

All analyses were performed using SAS version 9.4 (SAS Institute Inc., North Carolina, USA). Descriptive statistics were performed according to the type of variable. Quantitative variables were reported as the number of observed values, the number of missing values, the mean, the standard deviation (SD), the median, the minimum and maximum, and the first and third quartiles. Qualitative variables were reported as the number of observed values (frequencies), the number of missing values, and percentages.

Point estimates and appropriate two-tailed 95% CI were presented for the variables of interest. The 95% CI was provided for the primary and secondary endpoints. The 95% Wald CI of the mean was computed for quantitative parameters. For qualitative variables, the 95% CI calculation was based on the Agresti-Coull method. For inter-group comparison, a Student t-test or a Wilcoxon/Mann-Whitney test, depending on the normality of the data, was used to test the hypothesis involving quantitative variables. When Student’s t-test was chosen (in case of normality in all groups), a test of equality of variance was run to decide if a Satterthwaite’s correction must be applied; otherwise, the non-parametric Wilcoxon/Mann-Whitney test is used, and the W-value is reported. The Chi-square test or Fisher’s exact test, depending on the counts, was used to test the hypothesis involving qualitative variables. One-way analysis of variance (ANOVA) was considered to examine associations between the variables of interest: Defined age groups (four categories) and ADAMTS-13 enzymatic activity (three categories).

## Results

Characteristics of subjects whose samples were analyzed for ADAMTS-13 enzymatic activity

This study collected data from 463 samples obtained from subjects with known or suspected TTP, but the final number of analyzable samples was 390. Excluded samples came from subjects who were below 18 years of age or were of low quality, resulting in unavailable enzymatic activity. Originally, samples from 300 subjects in the Arabian Gulf were considered for analysis, residing in Saudi Arabia (N=293, of whom 214 were Saudis), Kuwait (N=4), and the United Arab Emirates (N=3). However, a total of 65 subjects were excluded. In Colombia, 163 samples were included, of which 155 were eligible for analysis. The overall age was 43.8±16.1 years (95% CI 42.2-45.4), with a median of 40 and a range of 18 to 90 years. There were 160 (41.0%) samples from men and 230 (59.1%) from women. Table 1 below reports on the age and gender of subjects whose samples were analyzed in this study.

**Table 1:** Demographic characteristics of the subject population CI: confidence interval; Max: maximum; Min: minimum; SD: standard deviation; Q: quartile.

	Arabian Gulf		Colombia	
	N=235	95% CI	N=155	95% CI
Age, in years				
Mean ± SD	41.3±13.6	39.6-43.0	47.7±18.7	44.8-50.7
Median (min-max)	38 (18-82)		45 (19-90)	
Q1-Q3	32-48		32-63	
Significance	Wilcoxon/Mann-Whitney W-value=33754.0 and P<0.01			
Categories, n (%)				
18-32 years	56 (23.8%)	18.8%-29.7%	40 (25.8%)	19.5%-33.3%
32-40 years	75 (31.9%)	26.3%-38.1%	19 (12.3%)	7.9%-18.4%
40-55 years	64 (27.2%)	21.9%-33.3%	39 (25.2%)	19.0%-32.6%
≥ 55 years	40 (17.0%)	12.7%-22.4%	57 (36.8%)	29.6%-44.6%
Significance	Test of independence X^2^=29.9246 and P<0.001			
Male gender, n (%)	100 (43.0%)	36.4%-49.0%	60 (38.7%)	31.4%-46.6%

Analysis of ADAMTS-13 enzymatic activity

ADAMTS-13 enzymatic activity was reported in samples obtained from a total of 390 subjects. Levels ranged between 0.1% and 142.2%, with a median of 45.7% and average of 42.8±31.1 (95% CI 39.8%-45.9%) ADAMTS-13 activity level was described in categories: TTP diagnosis (activity <10%) in 91 (23.3%), borderline ADAMTS-13 activity (levels between 10 and 20%) in 17 (4.4%) and absence of TTP (activity >20%) in 282 (72.3%). In the <10% category, enzymatic activity was 1.7%±1.9 (95% CI 1.3%-2.1%), with a median of 1.0%. In the 10-20% category, enzymatic activity was 15.4%±3.0 (95% CI, 13.8%-16.9%), with a median of 14.9%. In cases with ADAMTS-13 activity ≥10% (i.e., for whom a TTP diagnosis was not retained), enzymatic activity was 55.4%±24.2 (95% CI 52.6%-58.1%, ranging from 10.9% to 142.2%), with a median of 54.0%. The enzymatic activity level was compared between the Arabian Gulf region and Colombia. Specifically, in the Arabian Gulf, the average enzymatic activity was 39.9%±30.2, compared to 47.3%±32.0 in Colombia (Wilcoxon/Mann-Whitney W-value=32087.5, P=0.101), as shown in Table 2.

**Table 2:** ADAMTS-13 enzymatic activity levels ADAMTS-13: a disintegrin and metalloproteinase with a thrombospondin type 1 motif, member 13; CI: confidence interval; Max: maximum; Min: minimum; SD: standard deviation; Q: quartile.

	Arabian Gulf N=235	Colombia N=155
ADAMTS-13 activity level, in %		
Mean ± SD	39.9±30.2	47.3±32.0
95% CI	36.1-43.8	42.2-52.3
Median (min-max)	44.0 (0.1-122.0)	48.1 (0.2-142.2)
Q1-Q3	4.0-60.3	22.1-69.2
Significance	Wilcoxon/Mann-Whitney W-value=32087.5, P=0.101	
ADAMTS-13 activity category		
<10%, n (%); 95% CI	63 (26.8%); 21.5%-32.8%	28 (18.1%); 12.8%-24.9%
10-20%, n (%); 95% CI	11 (4.7%); 2.5%-8.3%	6 (3.9%); 1.6%-8.4%
>20%, n (%); 95% CI	161 (68.5%); 62.3%-74.1%	121 (78.1%); 70.9%-83.9%

**Figure 1:**
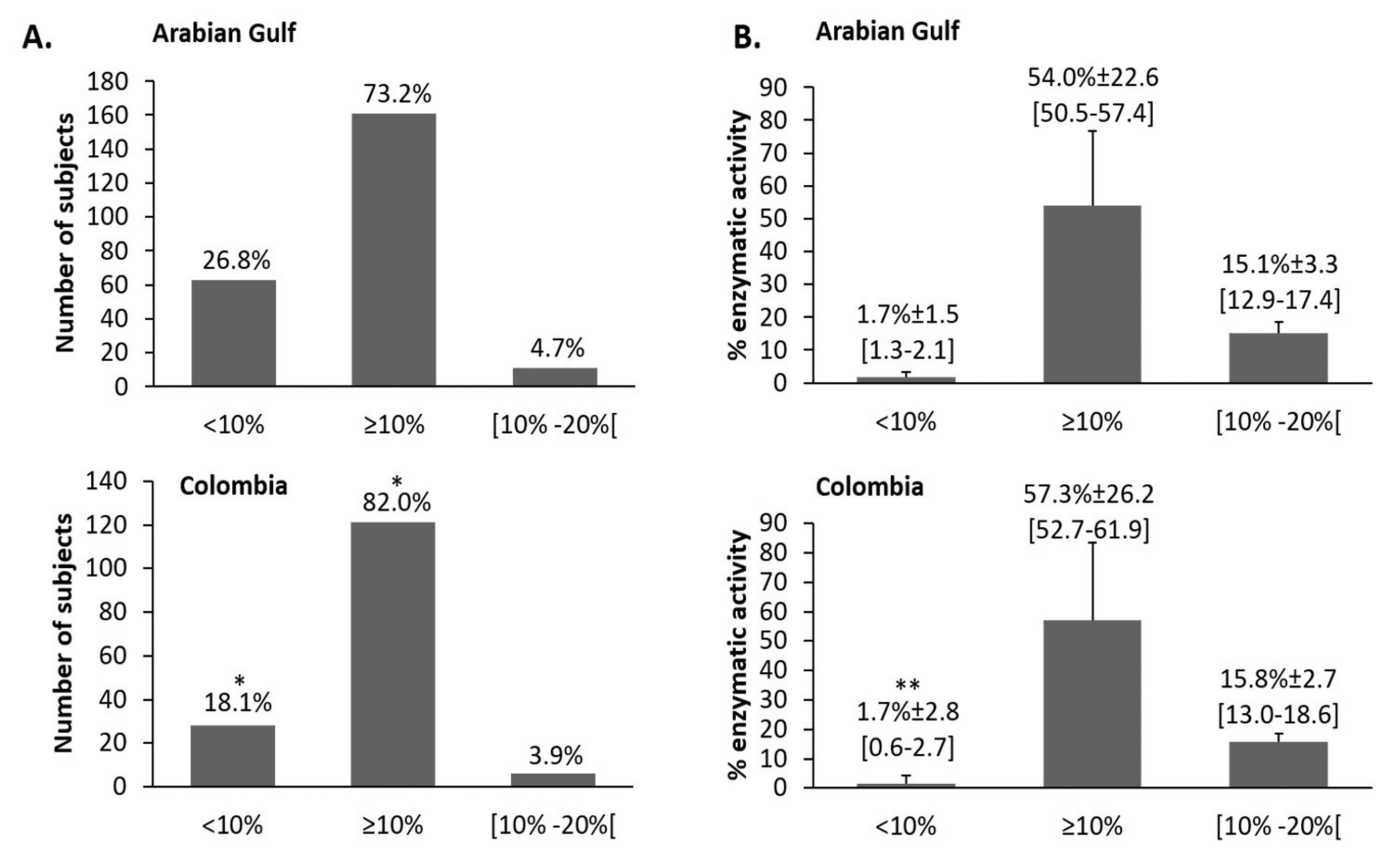
Distribution of subjects in the ADAMTS-13 enzymatic activity categories in the Arabian Gulf and in Colombia A. Proportion of subjects in the different ADAMTS-13 enzymatic activity levels: subjects with enzymatic activity levels <10% had severe ADAMTS-13 deficiency, indicating a TTP diagnosis, and subjects with enzymatic activity levels ≥10% do not qualify for a TTP diagnosis, although levels of 10-20% reflect borderline activity. B. Levels of ADAMTS-13 enzymatic activity in the different categories [given as mean ± standard deviation with (95% confidence interval)]. The P value compares data obtained in the Arabian Gulf to data from Colombia; specifically, results are compared between the Arabian Gulf (upper graph) and Colombia (lower graph), for the same outcome analyzed within each panel (A or B) *test of independence x^2^=3.991 and P<0.05 and **Wilcoxon/Mann-Whitney W-value=871.5 and P<0.001.

Figure 1 displays the distribution of subjects in three categories: ADAMTS-13 activity levels <10% (TTP diagnosis), ADAMTS-13 activity levels ≥10% (absence of TTP diagnosis), of which ADAMTS-13 activity levels ≥10% and <20% (suggestive of borderline ADAMTS-13 activity), in the Arabian Gulf and in Colombia. There was a significantly higher proportion of samples in the Arabian Gulf [63 (26.8%)] with ADAMTS-13 activity level <10%, compared to Colombia (28 [18.1%]), test of independence x^2^=3.991 and P=0.046 and significantly lower activity level in the category of severe ADAMTS-13 deficiency in the Arabian Gulf (Wilcoxon/Mann-Whitney W-value=871.5 and P<0.001).

Profile of subjects with TTP

In total, severe ADAMTS-13 deficiency, indicative of a TTP diagnosis, was reported in samples from 91 subjects. The average age of the subjects was 41.4±13.5 years, ranging from 18 to 81 years, with a median of 39 years. As shown in Table 3, this subject group had a lower average age than subjects with borderline ADAMTS-13 activity (49.4±16.4 years) or those with ADAMTS-13 activity levels greater than 20% (44.3±16.8 years, ANOVA F-score=1.6918 and P=0.1854). Among those subjects with TTP, there were 48 females (52.8%) and 43 males (47.3%), but sex distribution was not statistically significant (test of independence x^2^=0.3135 and P=0.576). None of the samples corresponding to subjects with TTP came from Kuwait; 28 (30.8%) came from Colombia, and 63 (69.2%) from Saudi Arabia.

**Table 3:** Demographic characteristics of subjects with TTP ADAMTS-13: a disintegrin and metalloproteinase with a thrombospondin type 1 motif, member 13; ANOVA: analysis of variance; SD: standard deviation; TTP: thrombotic thrombocytopenic purpura.

	Subjects with TTP, N=91	Subjects with borderline ADAMTS-13 activity, N=17	Subjects with ADAMTS-13 activity >20%, N=282
Age (Mean±SD), in years	41.4±13.5	49.4±16.4	44.3±16.8
ANOVA	F-score=1.6918 and P=0.1854		
Male subjects	43 (47.3%)	8 (47.1%)	109 (38.7%)
Female subjects	48 (52.8%)	9 (52.9%)	173 (61.3%)
Test of independence	X^2^=0.3135 P=0.576	X^2^=0.0322 P=0.8576	X^2^=0.1911 P=0.6620

Result turnaround time

Time from sample collection to laboratory results was 4.4±5.9 days, with a median of 2.0 days (Table 4). The time from sample collection to results was reported per region, and shows a significant difference between the Arabian Gulf countries (average of 6.5±6.9 days, 95% CI 5.6-7.4, with a median of 3.1 days) and Colombia (average of 1.2±0.8 days, 95% CI 1.1-1.4, with a median of 1.1 day, Wilcoxon/Mann-Whitney W-value=18507.5 and P<0.001). Additionally, the longest turnaround times in Colombia and the Arabian Gulf were 4.1 days and 45 days, respectively. Specifically, in the Arabian Gulf, 25% of samples were associated with a turnaround time that exceeded 11.9 (Q3) days. The turnaround time of these 58 samples in the Arabian Gulf was distributed as follows: >14 days (n=39), >21 days (n=7), and >28 days (n=3).

**Table 4:** ADAMTS-13 activity results turnaround time CI: confidence interval; Max: maximum; Min: minimum; SD: standard deviation; Q: quartile.

	Arabian Gulf N = 235	Colombia N = 155
Mean ± SD, in days	6.5 ± 6.9	1.2 ± 0.8
95% CI	5.6 - 7.4	1.1 - 1.4
Median (min-max)	3.1 (0.1 – 45.0)	1.1 (0.02 - 4.1)
Q1 - Q3	2.0 - 11.9	0.8 - 1.5

Significance.	Wilcoxon/Mann-Whitney W-value=18507.5 and P<0.001	

## Discussion

This study aimed primarily at filling the literature gap on reporting ADAMTS-13 enzymatic activity levels in adult subjects with suspected/confirmed TTP and determining the proportion of patients with a TTP diagnosis (ADAMTS-13 activity levels <10%) and those who do not have TTP (ADAMTS-13 activity levels ≥10%), according to the ISTH [30], in countries of the Arabian Gulf and in Colombia. Additionally, the turnaround time from sample collection to results (samples processed either in a local laboratory or a foreign laboratory) and subject characteristics were reported. Among the 390 samples from subjects eligible for analysis between Jan 2021 and Dec 2023, ADAMTS-13 enzymatic activity level was 42.8%±31.1. A total of 91 samples (23.4%) belonged to subjects diagnosed with TTP (severe ADAMTS-13 activity deficiency) and 17 (4.4%) to subjects with borderline enzymatic activity [i.e., ADAMTS-13 activity levels (10% and 20%)]. Notably, although activity levels <10% are considered diagnostic for TTP, borderline activity may be equivocal, per the ISTH's recent guidance [30]. Among subjects for whom a TTP diagnosis was not retained, ADAMTS-13 activity was 55.4% ± 24.2, ranging from approximately 11% to 142%. In the literature, some laboratories consider ADAMTS-13 activity >70% as normal [36], while others report that an activity between 50% and 160% is normal in healthy adults [37].

The vast majority of cases in the Arabian Gulf originated from Saudi Arabia, with 70% concentrated in the cities of Jeddah and Riyadh. In Colombia, 20.1% of cases were from Bogotá. A slightly higher proportion of samples had severe TTP with ADAMTS-13 enzymatic activity level <10% in the Arabian Gulf (26.8%), compared to Colombia (18.1%, test of independence X^2^=3.991 and P=0.046). In this study, 230 samples came from female subjects and 159 from male subjects, but an ADAMTS-13 enzymatic activity level <10% and a subsequent TTP diagnosis (in 48 female and 43 male subjects) were not significantly associated with gender. In most populations, TTP is presented with a female predilection, with a female-to-male ratio of 2:1 to 3:1 [6,7,38]. In this study, the median age at diagnosis (i.e., among subjects with ADAMTS-13 enzymatic activity level <10%) was 39 years, in line with the literature [6,38].

Of special relevance to potentially life-threatening diseases, the turnaround time for results was 4.4±5.9 days, with a median of 2.0 days. In the Arabian Gulf, 75% of samples had a turnaround time of less than 11.9 days; however, three samples took between 28 and 45 days to be analyzed. In Colombia, the longest turnaround time was 4.1 days. Cleveland Clinic laboratories estimate result turnaround within two to four days [36]. The turnaround time was significantly different between samples from the Arabian Gulf (average of 6.5±6.9 days) and those from Colombia (average of 1.2±0.8 days, Wilcoxon/Mann-Whitney W-value=18507.5 and P<0.001). Globally, the turnaround time for ADAMTS-13 activity level is estimated to be several days, which delays a definitive diagnosis and leads to non-diagnostically supported initial management decisions [6,30]. This study suggests that close to one quarter of samples tested for TTP diagnosis might have a confirmed severe deficiency in ADAMTS-13 activity and another 5% borderline activity, requiring pharmacological treatment for TTP (such as monoclonal antibody therapy with caplacizumab). This large proportion of patients might require life-saving medicines, and delays in test results might constitute an obstacle to therapeutic efficiency, leading to organ damage and perhaps death [1,3,4,32,33,39]. In fact, treatment for TTP is usually administered every one to three weeks, which means that if the diagnosis is delayed (also considering the lead time between symptom onset and seeking medical care), delays might be very detrimental to the patient [40,41]. In a 2023 study in Colombia, 10.4 ± 5.9 days went by on average between symptom onset and a definitive TTP diagnosis [42]. In the current study, most results were reported within 12 days, but longer delays might result in delayed treatment start, prompting investigation and action. While not corroborated in this study, potential reasons for prolonged turnaround times could include medical courier service delays, kit/laboratory supply delays, and waiting for samples to send to the central laboratory in one package or to run collectively on the same 96-well ELISA plate.

Limitations and strengths

This study did not utilize tools such as the PLASMIC score to identify subjects at higher risk for TTP, which prevented the correlation of clinical features of TTP with ADAMTS-13 activity levels in this population. Additionally, the data analyzed were exclusively from laboratory records, with no reports on clinical signs and symptoms or subsequent management. In fact, blood samples taken immediately following plasma therapy may yield a falsely elevated ADAMTS-13 activity [43], leading to an underestimation of the rate of ADAMTS-13 enzymatic activity deficiency and the rate of TTP cases in the study population. However, these limitations do not compromise the study outcomes. Moreover, this study secured a total of 390 samples, a major increase from the initially expected sample size of 140 samples. Although samples were analyzed in one laboratory in the Arabian Gulf and in two laboratories in Colombia, samples came from different cities. Despite the fact that most samples came from the two major Arab cities (Jeddah and Riyadh) and from major cities in Colombia, we expect the study population to be well-represented.

## Conclusions

TTP is a rare disease with life-threatening consequences. The results of this study underscore the need for more comprehensive evaluation of ADAMTS-13 enzymatic activity in subjects with clinical signs and symptoms indicative of TTP, for accurate diagnosis and timely treatment. The turnaround time in Colombia appears acceptable, but delays in obtaining a definite diagnosis are reported in the Arabian Gulf countries. This underscores the necessity of determining the reasons behind test result delays and upscaling the diagnostic process in the Arabian Gulf to prevent occasional result turnaround delays.
